# Reduction-Induced Suppression of Electron Flow (RISE) Is Relieved by Non-ATP-Consuming Electron Flow in *Synechococcus elongatus* PCC 7942

**DOI:** 10.3389/fmicb.2018.00886

**Published:** 2018-05-07

**Authors:** Ginga Shimakawa, Keiichiro Shaku, Chikahiro Miyake

**Affiliations:** ^1^Department of Biological and Environmental Science, Faculty of Agriculture, Graduate School of Agricultural Science, Kobe University, Kobe, Japan; ^2^Core Research for Environmental Science and Technology, Japan Science and Technology Agency, Tokyo, Japan

**Keywords:** P700 oxidation, photosynthesis, reactive oxygen species, plastoquinone, Q-cycle

## Abstract

Photosynthetic organisms oxidize P700 to suppress the production of reactive oxygen species (ROS) in photosystem I (PSI) in response to the lower efficiency of photosynthesis under high light and low CO_2_ conditions. Previously, we found a positive relationship between reduction of plastoquinone (PQ) pool and oxidation of P700, which we named reduction-induced suppression of electron flow (RISE). In the RISE model, we proposed that the highly reduced state of the PQ pool suppresses Q-cycle turnover to oxidize P700 in PSI. Here, we tested whether RISE was relieved by the oxidation of the PQ pool, but not by the dissipation of the proton gradient (ΔpH) across the thylakoid membrane. Formation of ΔpH can also suppress electron flow to P700, because acidification on the luminal side of the thylakoid membrane lowers oxidation of reduced PQ in the cytochrome *b*_6_/*f* complex. We drove photosynthetic electron transport using H_2_O_2_-scavenging peroxidase reactions. Peroxidase reduces H_2_O_2_ with electron donors regenerated along the photosynthetic electron transport system, thereby promoting the formation of ΔpH. Addition of H_2_O_2_ to the cyanobacterium *Synechococcus elongatus* PCC 7942 under low CO_2_ conditions induced photochemical quenching of chlorophyll fluorescence, enhanced NADPH fluorescence and reduced P700. Thus, peroxidase reactions relieved the RISE mechanism, indicating that P700 oxidation can be induced only by the reduction of PQ to suppress the production of ROS in PSI. Overall, our data suggest that RISE regulates the redox state of P700 in PSI in cooperation with ΔpH regulation.

## Introduction

Oxygenic phototrophs adjust photon energy utilization to environmental conditions in an attempt to alleviate photo-oxidative damage. Solar photon energy often exceeds photosynthetic CO_2_ assimilation needs, which has the potential to overflow into O_2_ in photosystem I (PSI), thereby generating reactive oxygen species (ROS), including superoxide anion radical, hydroxyl radical, and singlet oxygen (Satoh, 1970; Sonoike, 1996; Cazzaniga et al., 2012; Sejima et al., 2014; Takagi et al., 2016b). Because of their high reactivity, ROS immediately obliterate PSI photochemical activity and the inactivated PSI takes days to weeks to recover (Kudoh and Sonoike, 2002; Zivcak et al., 2015). The photo-oxidative damage in PSI, derived from ROS high reactivity, can be easily induced by repetitive short-pulse illumination, which instantaneously fills the photosynthetic electron transport system with electrons (Sejima et al., 2014). The inactivation of PSI is suppressed if the reaction center chlorophyll (Chl) in PSI, P700, is kept oxidized (Sejima et al., 2014; Shimakawa et al., 2016b, 2017a; Takagi et al., 2017b). Photosynthetic organisms flexibly oxidize P700 in response to high light intensity and low CO_2_ conditions, in an attempt to suppress ROS production (Badger and Schreiber, 1993; Golding and Johnson, 2003; Miyake et al., 2005; Sejima et al., 2014; Shimakawa et al., 2016b, 2017a; Takagi et al., 2017b). The oxidation of P700 strictly indicates that the re-reduction of oxidized P700 by electrons from PSII is prevented, but here we use the simple term “P700 oxidation” for this physiological response. P700 oxidation is a universal strategy used by photosynthetic organisms to decrease the risk of ROS production by lowering the amount of ground state P700, the source of excess electrons and energy. That is why photo-oxidative damage in PSI rarely occurs.

Oxidation of P700 in PSI is regulated by a variety of molecular mechanisms (P700 oxidation system). These are categorized as either acceptor-side mechanisms, i.e., those which safely dissipate excess electrons and energy through electron transport in order to relax the limitation of the electron acceptor side of PSI (alternative electron transport), or donor-side mechanisms, i.e., those which suppress electron transport into PSI (Shimakawa et al., 2016b, 2017a; Takagi et al., 2017b). In the case of acceptor-side mechanisms, photorespiration prepares a major alternative electron sink in land plants, except for C_4_ plants (Takagi et al., 2016a; Hanawa et al., 2017). Furthermore, flavodiiron protein mediates alternative electron transport to oxidize P700 in cyanobacteria (Helman et al., 2003; Allahverdiyeva et al., 2013; Shimakawa et al., 2015, 2016b), chlorophytes (Chaux et al., 2017), bryophytes (Gerotto et al., 2016; Shimakawa et al., 2017a), and probably pteridophytes and gymnosperms (Zhang et al., 2009; Takagi et al., 2017b). Both P700 oxidation systems on the electron acceptor side require O_2_ as the electron acceptor (Helman et al., 2003; Hayashi et al., 2014; Sejima et al., 2016; Hanawa et al., 2017). On the donor side, P700 oxidation is known to have a strong relationship with the proton gradient (ΔpH) across the thylakoid membrane. Studies on isolated chloroplasts have shown that acidification on the luminal side of the thylakoid membrane suppresses electron transport in the cytochrome *b*_6_/*f* complex (Cyt *b*_6_/*f*) (Tikhonov et al., 1981; Nishio and Whitmarsh, 1993). This has been subsequently supported by *in vivo* physiological measurements on intact plant leaves (Takizawa et al., 2008; Rott et al., 2011; Takagi et al., 2017a) and living cyanobacterial cells (Trubitsin et al., 2003). Additionally, energy-dependent non-photochemical quenching (qE or qZ) is activated by ΔpH to dissipate excess photon energy as heat at photosystem II (PSII) in plants, algae, and cyanobacteria (Niyogi and Truong, 2013; Stamatakis and Papageorgiou, 2014; Ruban, 2016). Furthermore, H_2_O oxidation in PSII is inhibited at low pH on the luminal side of the thylakoid membrane (Krieger et al., 1993). These mechanisms help alleviate the pressure of electron transport on the donor side of PSI and contribute to P700 oxidation.

**FIGURE 1:**
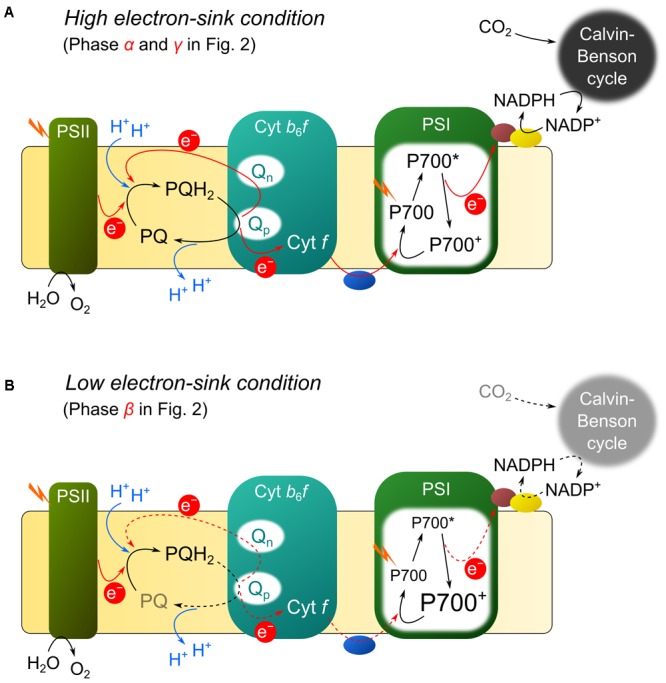
Proposed model of reduction-induced suppression of electron flow (RISE). **(A)** Q-cycle in high electron-sink conditions (i.e., *α* and *γ* in **Figure 2**). The activity of photosynthetic linear electron flow (LEF) is high. Plastoquinone (PQ) is reduced to plastoquinol (PQH_2_) with electrons from photosystem II (PSII) and the Q_n_ site in the cytochrome *b*_6_/*f* complex (Cyt *b*_6_/*f*). Then, PQH_2_ is oxidized at the Q_p_ site in Cyt *b*_6_/*f*. **(B)** The Q-cycle in low electron-sink conditions (i.e., *β* in **Figure 2**). The activity of LEF is low, and PQ pool is highly reduced, leading the suppression of the Q cycle in Cyt *b*_6_/*f* to oxidize P700 in photosystem I (PSI). Blue, brown, and yellow ellipsoid respectively indicate plastocyanin (or cytochrome *c*_6_), ferredoxin, and ferredoxin-NADP^+^ reductase. Red and blue lines indicate the transports of electrons and protons, and dashed red line shows the suppressed electron flow.

Recently, Shaku et al. (2016) identified a novel P700 oxidation mechanism operating on the donor side of PSI: reduction-induced suppression of electron flow (RISE). In photosynthetic linear electron flow (LEF) on the thylakoid membrane, plastoquinol (PQH_2_) is oxidized to plastoquinone (PQ) in Cyt *b*_6_/*f*, where the Q-cycle operates (**Figure 1**; Kallas, 1994; Tikhonov, 2014). In the Q-cycle, PQH_2_ donates one electron to an iron-sulfur cluster at the PQH_2_ oxidation site (Q_p_ site) in Cyt *b*_6_/*f*; cytochrome *f* (Cyt *f*) accepts the electron from the iron-sulfur cluster. The electron in the PQ semiquinone remaining at the Q_p_ site is transferred to a PQ at the PQ reduction site (Q_n_ site) in Cyt *b*_6_/*f*. The PQ in the one electron-reduced form at the Q_n_ site accepts the second electron from PSII and becomes reduced to PQH_2_ at the Q_n_ site in Cyt *b*_6_/*f*. When two molecules of PQH_2_ are oxidized at the Q_p_ site in Cyt *b*_6_/*f*, two electrons are transported to Cyt *f* sequentially and the other two circulate within Cyt *b*_6_/*f* to produce one molecule of PQH_2_ at the Q_n_ site (**Figure 1**; Kallas, 1994; Tikhonov, 2014). Theoretically, unless PQ is supplied to the Q_n_ site, the Q-cycle cannot operate and the reduction of Cyt *f* is suppressed. Shaku et al. (2016) showed that a reduction of the PQ pool in the *Synechococcus elongatus* PCC 7942 (*S*. *elongatus*) flavodiiron protein-deficient mutant causes suppression of electron transport from PQH_2_ to PSI, which in turn results in the accumulation of oxidized P700. That is, in the mutant, the Q-cycle function is suppressed due to the shortage of PQ supplied for the Q_n_ site in Cyt *b*_6_/*f*, resulting in suppressed LEF under CO_2_ limitation. Therefore, the mutant can survive in an air-equilibrated condition (Shimakawa et al., 2016b).

As described above, on the electron donor side of PSI, two molecular mechanisms for P700 oxidation can function: the ΔpH-dependent suppression of PQH_2_ oxidation in Cyt *b*_6_/*f* and the suppression of the Q-cycle, which depends on the accumulation of PQH_2_ (RISE). In order to demonstrate that RISE is regulated by electron-sink activity, in the present study, we tested whether RISE is relieved by a non-ATP-consuming metabolic pathway. A previous report by Shaku et al. (2016) showed that P700 oxidation induced by RISE is suppressed by addition of NaHCO_3_ to the cyanobacterial cells. Simultaneously, NaHCO_3_ starts CO_2_-dependent O_2_ evolution, i.e., photosynthesis. Carbon assimilation consumes ATP in addition to NADPH and dissipates ΔpH formed across the thylakoid membrane. Based on these facts, we could not exclude the possibility that the suppression of P700 oxidation might be driven by the increased activity of Cyt *b*_6_/*f* under conditions of dissipated ΔpH. Thus, we tried to show that a non-ATP-consuming metabolic pathway, electron sink, prevents RISE from operating. We investigated the effect of H_2_O_2_-dependent electron flow as a non-ATP-consuming metabolic pathway on the relaxation of RISE in *S. elongatus*.

Cyanobacteria detoxify H_2_O_2_ using catalase and peroxidase reactions (Miyake et al., 1991). The peroxidase reaction uses electron donors such as NADPH (Miyake et al., 1991; Yamamoto et al., 1999). For continuous scavenging of H_2_O_2_, these electron donors are regenerated by LEF (Miyake et al., 1991). Therefore, addition of H_2_O_2_ to cyanobacterial cells induces both photochemical quenching of Chl fluorescence and O_2_ evolution in the light (Miyake et al., 1991). That is, H_2_O_2_-dependent peroxidase reaction drives LEF, which is also observed in isolated intact chloroplasts from plant leaves (Schreiber and Neubauer, 1990; Miyake and Asada, 1992). This H_2_O_2_-dependent peroxidase reaction does not consume ATP. Therefore, if the H_2_O_2_-dependent peroxidase reaction results in RISE shutting off, then it would indicate that electron sink activity regulates RISE.

The NADPH redox level provides the information of the dynamic property of the electron acceptor side of PSI, which can be evaluated as blue green fluorescence using a Dual-PAM-100 instrument (Heinz Walz, Effeltrich, Germany; Mi et al., 2000; Schreiber and Klughammer, 2009; Kauny and Sétif, 2014; Holland et al., 2015; Shaku et al., 2016). Recently, Holland et al. (2015) investigated the dynamic response of the NADPH redox level to CO_2_ limitation in the cyanobacterium *Synechocystis* sp. PCC 6803. Limiting CO_2_ causes the suppression of the Calvin-Benson cycle to lower the efficiency of the consumption of NADPH, resulting in the reduction of the NADP^+^ pool (Holland et al., 2015). However, the NADP^+^ pool is not fully reduced even under CO_2_ limitation, indicating that not only the consumption but also the production of NADPH is suppressed in response to CO_2_ limitation. Overall, the abovementioned molecular mechanisms for P700 oxidation contribute to keep part of the NADP^+^ pool oxidized, and it is expected that the pool will be more reduced when the suppression of electron transport in Cyt *b*_6_/*f* is relaxed.

## Materials and Methods

### Growth Conditions and Chl *a* Determination

Cyanobacterial cultures were maintained on BG-11 solid medium (Allen, 1968) under continuous fluorescent lighting (25°C, 50 μmol photons m^-2^ s^-1^). For all physiological experiments, cells from the cultures were inoculated into BG-11 liquid medium (initial OD_750_: 0.1–0.2) and grown on a rotary shaker (100 rpm) under a light/dark cycle (light period: 16 h, at 25°C, 150 μmol photons m^-2^ s^-1^; dark period: 8 h, at 23°C), at 2% (v/v) [CO_2_]. Optical density of the medium at 750 nm was measured with a spectrophotometer (U-2800A, Hitachi, Tokyo, Japan). Cells from the early exponential growth phase (OD_750_: 2–3) were used for the experiments.

For Chl measurements, cells from 0.1 to 1.0 mL cultures were harvested by centrifugation and resuspended by vortexing in 1 mL 100% (v/v) methanol. After incubation at room temperature for 5 min, the suspension was centrifuged at 10,000 × *g* for 5 min. Total Chl *a* was spectrophotometrically determined from the supernatant (Grimme and Boardman, 1972).

### Measurement of Chl and NADPH Fluorescence, and P700 Absorbance

Both Chl and NADPH fluorescence were simultaneously measured with a Dual-PAM-100 instrument (Heinz Walz, Effeltrich, Germany) at room temperature (25°C ± 2°C). The reaction mixtures (2 mL) contained 50 mM HEPES (pH 7.5) and the cells (10 μg Chl mL^-1^). During the measurement, the reaction mixture was stirred with a magnetic micro stirrer. Photon flux density of red actinic light (AL, LED with peak emission at 635 nm) was 200 μmol photons m^-2^ s^-1^. The values of incident quantum yield of PSII, Y(II), which reflect the apparent electron flux in LEF (Genty et al., 1989; Shimakawa et al., 2017b), were calculated from Chl fluorescence as (F_m_′ – F_s_)/F_m_′: F_m_′, maximum variable fluorescence yield; F_s_, steady-state fluorescence yield; and F_o_, minimum fluorescence yield (Schreiber et al., 1986; van Kooten and Snel, 1990). A 300 ms saturation pulse light (LED with peak emission at 635 nm, 10,000 μmol photons m^-2^ s^-1^) was supplied for the determination of F_m_′.

The NADPH fluorescence originated in NAD(P)H was measured using the NADPH/9-AA module of a Dual-PAM-100 instrument (Heinz Walz, Effeltrich, Germany; Mi et al., 2000; Schreiber and Klughammer, 2009; Kauny and Sétif, 2014). The NADPH/9-AA module consists of an emitter unit (DUAL-ENADPH) and a detector unit (DUAL-DNADPH). NADPH fluorescence was excited by UV-A (365 nm) from the DUAL-ENADPH unit and detected by a blue-sensitive photomultiplier with a filter transmitting light between 420 and 580 nm in the DUAL-DNADPH unit. The measuring light intensity was on a scale from 1 to 20, and the intensity was set at 20 in this study. The measuring light frequency in the absence and presence of red AL was set at 200 and 5,000 Hz, respectively. We followed Schreiber and Klughammer (2009) for using the terms of NADPH fluorescence parameters: N_m_, the signal level for fully reduced NADP^+^ pool; N_o_, the signal level for fully oxidized NADP^+^ pool; N_t_, the current signal for the relative extent of NADP^+^ reduction.

Measurement of P700 absorbance was performed with a Dual-PAM-100 instrument (Heinz Walz, Effeltrich, Germany) in almost the same conditions as described for Chl and NADPH fluorescence analysis. The redox state of P700 was determined according to the method of Klughammer and Schreiber (2008). In this procedure, P_m_ = maximum P700 photo-oxidation level, obtained by a saturated pulse light under far-red illumination; P = oxidation level of P700 under AL; P_m_′ = maximum oxidation level of P700, obtained by a saturation pulse under AL illumination; Y(I) = (P_m_′ - P)/P_m_ = incident quantum yield of photochemical energy conversion; Y(ND) = P/P_m_ = quantum yield of non-photochemical energy dissipation due to a donor-side limitation and Y(NA) = (P_m_ - P_m_′)/P_m_ = quantum yield of non-photochemical energy dissipation due to an acceptor-side limitation. The sum of the three factors [Y(I) + Y(NA) + Y(ND)] = 1. For the determination of these parameters, a 300 ms saturation pulse (10,000 μmol photons m^-2^ s^-1^) was used, and the stirrer was turned off 5 s before the saturation pulse was applied.

### Measurement of O_2_ Exchange

Uptake and evolution of O_2_ were measured with a Clark-type O_2_ electrode at 25°C (Hansatech, King’s Lynn, United Kingdom) with a high time resolution (Sejima et al., 2016; Hanawa et al., 2017). The O_2_ amount in the reaction mixture were obtained in an analog recorder with the signal amplitude and the time scale properly adjusted as in Shimakawa et al. (2016a). The reaction mixture (2 mL) contained 50 mM HEPES (pH 7.5) and the cyanobacterial cells (10 μg Chl mL^-1^). Red AL (620 < *λ* < 695 nm, 200 μmol photons m^-2^ s^-1^) was provided by a halogen lamp (Xenophot HLX 64625, Osram, München, Germany) with an LS2 light source (Hansatech, King’s Lynn, United Kingdom). During the measurement, the reaction mixture was stirred with a magnetic micro stirrer.

### Generation of Mutants

The *S. elongatus katG* deficient mutant (*Synpcc7942_1656*) was generated by the method of Shaku et al. (2016). To obtain the knock-out construct (Supplementary Figure S1A), polymerase chain reaction (PCR) was used to amplify the genomic region encoding *katG* with a primer set (f, TTCCAATTTTGCTGCGCTTA; r, GCATTCATCACCTTCGTCCA). The PCR product was then cloned into the pGEM-T Easy vector (Promega, Tokyo, Japan). The recombinant plasmid was linearized and amplified by inverse PCR with a primer set (f, TTGGGCTTCGGAATATGGCAGTGGGAACCGATTA; r, AAACCGCCCAGTCTAGACAGCGTTGCGACCAATAC), and then applied to the In-Fusion reaction (Takara, Shiga, Japan) with a kanamycin-resistance gene (*Kan^r^*) derived from pUC4K vector (Taylor and Rose, 1988; Shimakawa et al., 2015). Transformation of wild type *S. elongatus* was performed by the standard procedure (Williams, 1988), and the mutant, Δ*katG*, was selected on BG-11 agar plates containing kanamycin (20 μg mL^-1^). Complete segregation was confirmed by PCR (Supplementary Figure S1B).

## Results and Discussion

### Experimental Scheme

**FIGURE 2:**
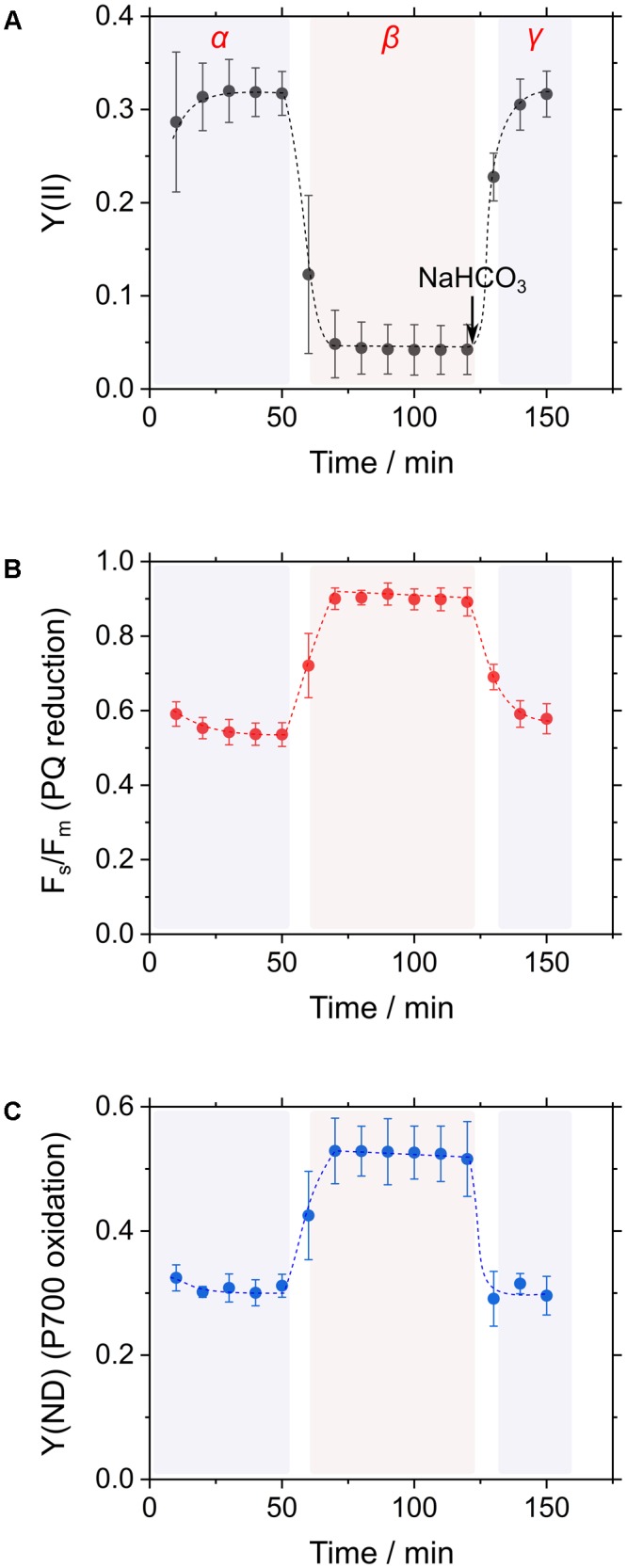
Time courses of the incident quantum yield of PSII, Y(II) **(A)**, the chlorophyll fluorescence parameter inferring the reduction of the plastoquinone (PQ) pool, F_s_/F_m_ **(B)**, and the electron donor-side limitation of photosystem I, Y(ND) **(C)** in response to CO_2_ limitation in *Synechococcus elongatus* PCC 7942. Cyanobacterial photosynthesis starts at 0 min with an actinic light. Thereafter, Y(II) decreases due to a shortage of CO_2_, which is defined as the transition from phase *α* to *β*. The addition of CO_2_ in the form of NaHCO_3_ restores photosynthetic activity (phase *γ*). Data are mean ± the standard deviation of three measurements (technical replicates).

In general, CO_2_ consumption under constant light suppressed cyanobacterial photosynthesis, as observed in the decrease in incident quantum yield of PSII, Y(II), which is estimated from Chl fluorescence analysis (**Figure 2A**; Hayashi et al., 2014; Shimakawa et al., 2015, 2016b). Addition of NaHCO_3_ to the cyanobacterial cells restored photosynthesis, as observed in the increase in Y(II) (**Figure 2A**; Hayashi et al., 2014; Shimakawa et al., 2015, 2016b). Experimentally, this can be observed as a three-phase (*α, β*, and *γ*; **Figure 2A**) time course. Reduction state of the PQ pool, reflected in F_s_/F_m_, responds to these three phases. In phase *α*, during which high photosynthetic rate is observed, PQ is oxidized, and in phase *β*, during which low photosynthetic rate is observed, PQ is reduced, as inferred from the increase in the Chl fluorescence parameter F_s_/F_m_ (**Figure 2B**; Hayashi et al., 2014). The reduced state of PQ is relieved in phase *γ*. Furthermore, the oxidation state of P700, reflected in quantum yield of non-photochemical energy dissipation due to donor-side limitation, Y(ND), from P700 absorbance analysis, also responds to these three phases in a similar fashion to the redox state of PQ (**Figure 2C**; Shaku et al., 2016; Shimakawa et al., 2016b). We refer to these responses of Y(II), F_s_/F_m_, and Y(ND) as RISE (Shaku et al., 2016).

We can explain RISE using the model of the Q-cycle, as shown in **Figure 1**. In phase *α* and *γ*, the Q-cycle operates in a high-electron sink condition (**Figure 1A**). The occupancy of the oxidized form of PQ is high, and an electron from the reduced form of PQ at the Q_p_ site in Cyt *b*_6_/*f* can be rapidly transferred into the Q_n_ site for the reduction of PQ; that is, the high-electron sink condition makes Q-cycle turnover rapid (**Figure 1A**). Conversely, in phase *β*, in which electron sink activity is low (i.e., low-electron sink condition; **Figure 1B**), Q-cycle turnover is slowed down. A low-electron sink condition reduces PQ, as inferred from the increase in F_s_/F_m_ (**Figure 2B**; Hayashi et al., 2014; Shaku et al., 2016); that is, the ratio of PQ to PQH_2_ decreases and the efficiency of the donation of electrons from Q_p_ to Q_n_ sites decreases. This results in the suppression of Q-cycle turnover, which in turn suppresses the reduction of cytochrome *f*, plastocyanin (or cytochrome *c*_6_), and eventually oxidizes P700 (**Figure 1B**). This is the mechanism for oxidation of P700 in phase *β* (**Figure 2C**). We refer to this modulation of Q-cycle turnover for P700 oxidation as RISE (Shaku et al., 2016).

In the present study, we aimed to further characterize the possible mechanism underlying RISE. In our previous report (Shaku et al., 2016), we activated photosynthesis with NaHCO_3_ to relax RISE. The activation of photosynthesis dissipates ΔpH across the thylakoid membrane by the consumption of ATP. The acidification of the lumen also suppresses the oxidation activity of PQH_2_ in Cyt *b*_6_/*f* (Tikhonov et al., 1981; Nishio and Whitmarsh, 1993), similar to RISE. We tried to relieve RISE by stimulating electron flow in phase *β* to prove that RISE is regulated by the redox state of PQ and the electron sink activity. We used H_2_O_2_-dependent electron flow (Miyake et al., 1991; Miyake and Asada, 1992). Cyanobacteria have several peroxidases, which utilize electron donors to reduce H_2_O_2_ to H_2_O (Miyake et al., 1991; Yamamoto et al., 1999). For continuous scavenging of H_2_O_2_, the oxidized electron donor is reduced by the photosynthetic electron transport system (Miyake and Asada, 1992). That is, addition of H_2_O_2_ to cyanobacterial cells drives LEF. The H_2_O_2_-dependent electron flow induces ΔpH across the thylakoid membrane because no ATP is consumed (Schreiber and Neubauer, 1990; Miyake and Asada, 1992).

To elucidate the occurrence of RISE and the response to the electron sink activity in *S. elongatus*, we simultaneously evaluated Chl and NADPH fluorescence after the establishment of phase *β* by the consumption of CO_2_ in the reaction mixture. In phase *β*, F_s_/F_m_ is kept at higher values (**Figure 2B**). Addition of an electron acceptor to the photosynthetic electron transport system should decrease F_s_/F_m_. The decrease in F_s_/F_m_ would show the acceleration of electron flow driven by the electron acceptor.

### Relaxing of RISE and Acceleration of Linear Electron Flow by Exogenous NaHCO_3_ in *S. elongatus*

**FIGURE 3:**
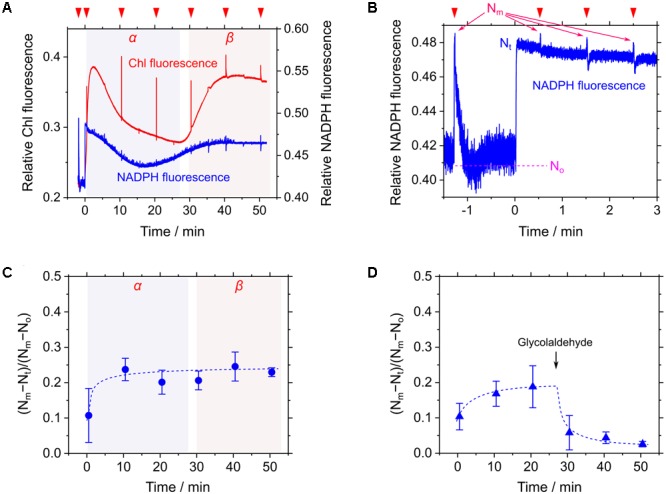
Responses of relative chlorophyll (Chl) and NADPH fluorescence to CO_2_ limitation in *Synechococcus elongatus* PCC 7942. **(A)** Time courses of relative Chl (red line) and NADPH (blue line) fluorescence during the transition from phase *α* to *β* of photosynthesis. Inverted red triangles show the application of the saturated short-pulse light. **(B)** The kinetics of NADPH fluorescence for the calculation of the oxidation fraction of NADP^+^ pool during the measurement. The use of the term N_m_, N_o_, and N_t_ are according to Schreiber and Klughammer (2009) (see “Materials and Methods”). The line graphs **(A,B)** show the representative results of three experiments (technical replicates). **(C,D)** Time courses of the oxidation fraction of NADP+ pool, (N_m_-N_t_)/(N_m_-N_o_), in the transition from phase *α* to *β* of photosynthesis **(C)** and in response to added 25 mM glycolaldehyde **(D)**. Data are mean ± the standard deviation of three experiments (technical replicates).

Cells of *S. elongatus* were illuminated with red AL (200 μmol photons m^-2^ s^-1^) without the supplement of an inorganic carbon source. Steady-state Chl fluorescence yield (i.e., F_s_) immediately increased in response to AL and then gradually decreased to a constant value during phase *α* (**Figure 3A**). Thereafter, F_s_ dramatically increased (**Figure 3A**), accompanying the decrease in Y(II) from 0.32 ± 0.04 at 20 min in phase *α* to 0.023 ± 0.003 at 40 min in phase *β* (mean ± standard deviation, *n* = 3). In this study, we sought to evaluate the NADPH redox level during the measurements following the method by Schreiber and Klughammer (2009). Because the base line signal of the NADPH fluorescence can drift during a long-term measurement (Schreiber and Klughammer, 2009; Kauny and Sétif, 2014; Holland et al., 2015), the maximum reduction level of NADP^+^ pool, defined as N_m_, was periodically determined by applying a saturated short-pulse light (1 s, 10,000 μmol photons m^-2^ s^-1^; **Figure 3B**). Additionally, the maximum oxidation level of NADP^+^ pool, defined as N_o_, was determined in the dark just after applying the short-pulse light (**Figure 3B**). The current NADPH fluorescence signal (N_t_) was continuously monitored. That is, the oxidation fraction of NADP^+^ pool was estimated as (N_m_-N_t_)/(N_m_-N_o_) during the measurements (Schreiber and Klughammer, 2009). During the transition to CO_2_ limitation (from phases *α* to *β*), we periodically determined (N_m_-N_t_)/(N_m_-N_o_), and found that the redox level of NADP^+^ pool did not change in response to CO_2_ limitation (**Figure 3C**). On the other hand, adding the Calvin-Benson cycle inhibitor glycolaldehyde caused the decrease in (N_m_-N_t_)/(N_m_-N_o_) (**Figure 3D**). These facts indicate that NADP^+^ pool is not fully reduced even under CO_2_ limitation, which is consistent with the preceding report (Holland et al., 2015). In response to CO_2_ limitation, LEF is suppressed in Cyt *b*_6_/*f* and P700 is kept oxidized (Shaku et al., 2016; Shimakawa et al., 2016b), which should lower the production of NADPH to save the oxidation fraction of NADP^+^ pool. On the other hand, it is expected that the more severe suppression of the Calvin-Benson cycle can cause the reduction of NADP^+^ pool, which is supported by the effect of glycolaldehyde on the NADPH fluorescence signal (**Figure 3D**; Holland et al., 2015). That is, the NADPH redox level severely depends on the degree of the suppression of the Calvin-Benson cycle, which might cause a different response of the NADPH redox level to CO_2_ limitation (Holland et al., 2015). In this study, we note that electron flow to the oxidized P700 in PSI was suppressed strongly enough not to reduce NADP^+^ in phase *β*. We refer to the suppressed electron flow to P700 in phase *β* as RISE.

**FIGURE 4:**
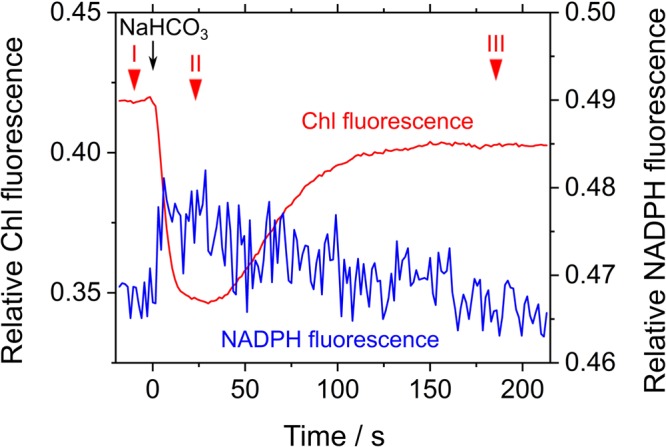
Effects of exogenous NaHCO_3_ on relative chlorophyll (Chl, red line) and NADPH (blue line) fluorescence in phase *β* in *Synechococcus elongatus* PCC 7942. Black arrow shows the point in time at which NaHCO_3_ (50 μM) was added. The incident quantum yield of PSII, Y(II), was measured at the points in time indicated by red inverted triangles: I, 0.028 ± 0.006; II, 0.12 ± 0.02; and III, 0.029 ± 0.007 (mean ± standard deviation, *n* = 3). The line graphs show the representative results of three experiments (technical replicates).

We evaluated the relaxation of RISE by adding NaHCO_3_ to the cells of *S. elongatus* in phase *β*. NaHCO_3_-dependent relief of RISE would be expected to increase the electron flow to NADP^+^ by oxidizing PQH_2_ and/or dissipating ΔpH for ATP synthesis. We added 50 μM NaHCO_3_ to *S. elongatus* in phase *β* and observed photochemical quenching reflected as a rapid decrease in F_s_ (**Figure 4**). That is, PQH_2_ was oxidized. We determined Y(II) at three points in time during the experiment (**Figure 4**): I, before NaHCO_3_ was added; II, while Chl fluorescence was photochemically quenched; and III, after F_s_ returned to a high level (0.028 ± 0.006, 0.12 ± 0.02, and 0.029 ± 0.007, respectively [mean ± standard deviation, *n* = 3]). The results showed that NaHCO_3_ enhanced electron flux in LEF, which led us to expect that stimulated photosynthetic CO_2_ assimilation would enhance NADPH consumption (Hayashi et al., 2014). Additionally, NADPH fluorescence rapidly increased by the addition of NaHCO_3_ and then gradually decreased (**Figure 4**). These results suggest that addition of NaHCO_3_ transiently reduced NADP^+^ and then gradually oxidized NADPH. As shown by the pattern of Chl fluorescence, PQH_2_ accumulated in phase *β* was oxidized to relieve RISE, which accelerated the electron flux to NADP^+^. Oxidation efficiency of NADPH in NaHCO_3_-stimulated photosynthesis was overwhelmed by the reduction efficiency of NADP^+^ in LEF, accelerated by the relaxing of RISE. This would explain why the oxidation of NADPH was not observed upon NaHCO_3_ addition to the cells. Overall, the addition of NaHCO_3_ relaxed RISE. However, we could not conclude whether the oxidation of PQH_2_ or the dissipation of ΔpH across the thylakoid membrane relaxed RISE.

### Relaxing of RISE and Acceleration of Linear Electron Flow by Exogenous H_2_O_2_ in *S. elongatus*

**FIGURE 5:**
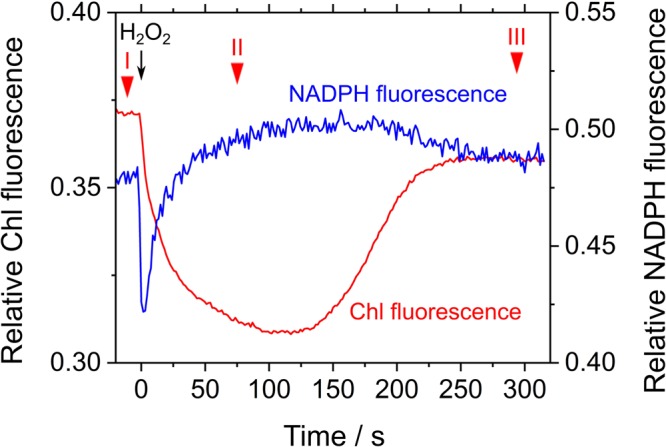
Effects of exogenous H_2_O_2_ on relative chlorophyll (Chl, red line) and NADPH (blue line) fluorescence in phase *β* in *Synechococcus elongatus* PCC 7942. Black arrow shows the point in time at which H_2_O_2_ (50 μM) was added. Catalase activity of the cyanobacterial cells was inhibited by adding hydroxylamine (25 μM). The incident quantum yield of PSII, Y(II), was measured at the points in time indicated by red inverted triangles: I, 0.027 ± 0.004; II, 0.120 ± 0.011; and III, 0.027 ± 0.007 (mean ± standard deviation, *n* = 3). The line graphs show the representative results of three experiments (technical replicates).

Next, we studied the effect of exogenous H_2_O_2_ on Chl and NADPH fluorescence in phase *β* in *S. elongatus*. The measurement was performed in the presence of hydroxylamine (25 μM), a catalase inhibitor. Upon addition of 50 μM H_2_O_2_, F_s_ decreased, which resulted in Y(II) values of 0.027 ± 0.004, 0.120 ± 0.011, and 0.027 ± 0.007 at points in time I, II, and III, respectively (mean ± standard deviation, *n* = 3) (**Figure 5**). In other words, photochemical quenching occurred in response to the addition of H_2_O_2_ in phase *β*. Thereafter, F_s_ increased with the consumption of H_2_O_2_ (**Figure 5**; Miyake et al., 1991). NADPH fluorescence immediately decreased in response to the addition of H_2_O_2_, and then gradually increased (**Figure 5**). The increase in NADPH fluorescence was accompanied by enhanced electron flux through LEF, as observed in the increase in Y(II). Thereafter, NADPH fluorescence decreased with the consumption of H_2_O_2_, as evidenced by the increase in F_s_ (**Figure 5**).

Simultaneously, we analyzed the response of the redox state of P700 in PSI to the addition of H_2_O_2_ to the cyanobacterial cells in phase *β*. Compared with the redox state of P700 at point I, the addition of H_2_O_2_ increased incident quantum yield of PSI, Y(I), and decreased Y(ND), at point II (**Table 1**). Thereafter, both Y(I) and Y(ND) recovered to the original values at point III (**Table 1**). Quantum yield of non-photochemical energy dissipation due to acceptor-side limitation Y(NA), did not show any changes (**Table 1**). These results indicate that in the scavenging of H_2_O_2_ the electron flux to NADP^+^ was enhanced, although P700^+^ was reduced, that is, the scavenging of H_2_O_2_ induced the oxidation of PQH_2_, which accompanied enhanced electron flux to NADP^+^ transiently. This shows that RISE was indeed relaxed only by the oxidation of PQH_2_, as observed in the decrease in Y(ND).

**Table 1:** Effects of exogenous H_2_O_2_ on the redox state of P700 in phase *β* in *Synechococcus elongatus* PCC 7942 (mean ± standard deviation, *n* = 3).

	Y(I)	Y(ND)	Y(NA)
I	0.36 ± 0.03	0.55 ± 0.01	0.09 ± 0.02
II	0.51 ± 0.03	0.37 ± 0.05	0.12 ± 0.02
III	0.34 ± 0.01	0.56 ± 0.02	0.10 ± 0.03

In cyanobacteria, H_2_O_2_ can be scavenged via two types of reactions, (i) catalase and (ii) peroxidase (Miyake et al., 1991; Tichy and Vermaas, 1999; Yamamoto et al., 1999; Stork et al., 2009):

(1) $$\begin{array}{c} H_{2}O_{2}\to 1/2O_{2}\;+\;H_{2}O \end{array}$$

(2) $$\begin{array}{c} H_{2}O_{2}\;+\;Red\cdot H_{2}\to 2H_{2}O\;+\;Oxi \end{array}$$

Catalase detoxifies H_2_O_2_ to H_2_O and O_2_ is evolved, whereas peroxidases utilize electron donors (indicated by *Red* and *Oxi* as the reduced and oxidized forms of electron donors). For example, in *S. elongatus*, thioredoxin functions as the electron donor in both thioredoxin peroxidase and peroxiredoxin Q reactions (Stork et al., 2009). Thioredoxin is reduced by NADPH-thioredoxin reductase with NADPH (iii) (Miyake et al., 1991; Yamamoto et al., 1999). The NADP^+^ produced in the peroxidase reactions is reduced back to NADPH in the photosynthetic electron transport system (iv); the scavenging of H_2_O_2_ by the peroxidase reactions is coupled with LEF, which is linked to the evolution of O_2_ in PSII (v) (Miyake et al., 1991; Yamamoto et al., 1999; Miyake and Asada, 2003).

(3) $$\begin{array}{c} Oxi\;+\;NADPH\;+\;H^{+}\to Red\cdot H_{2}\;+\;NADP^{+} \end{array}$$

(4) $$\begin{array}{c} H_{2}O\;+\;NADP^{+}\to 1/2O_{2}\;+\;NADPH\;+\;H^{+} \end{array}$$

(5) $$\begin{array}{c} H_{2}O_{2}\to 1/2O_{2}\;+\;H_{2}O \end{array}$$

Thus, exogenous H_2_O_2_ functions as the alternative electron acceptor to stimulate LEF, as supported by photochemical quenching of Chl fluorescence (**Figure 5**). The rapid decrease in NADPH fluorescence immediately after H_2_O_2_ addition might be due to consumption of NADPH via the abovementioned peroxidase reactions, with the accumulation of the oxidized form of the electron donors, NADPH fluorescence increased by the relaxation of RISE.

We conclude that some parts of electron transport suppression in phase *β* in *S. elongatus*, depend only on the redox state of the PQ pool, but not on ΔpH (**Figure 5**). Exogenous H_2_O_2_ accelerated LEF to reduce NADP^+^; the gradual increase in NADPH fluorescence was clearly related to the photochemical quenching of Chl fluorescence (**Figure 5**). From the above-mentioned formulae, peroxidase-dependent H_2_O_2_-scavenging reactions do not require ATP; ΔpH formation is rather promoted (Schreiber and Neubauer, 1990). In other words, the oxidized electron donor produced in the peroxidase reactions effectively relieved RISE.

**FIGURE 6:**
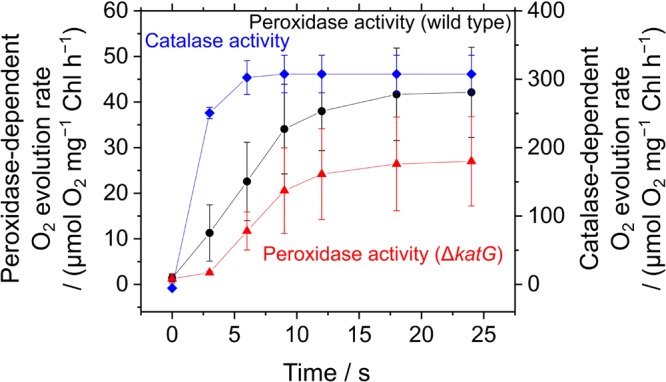
Induction of O_2_ evolution by exogenous H_2_O_2_ through peroxidase and catalase activities in *Synechococcus elongatus* PCC 7942. Peroxidase-dependent O_2_ evolution was measured in the presence of hydroxylamine (25 μM) in phase *β* in the wild type (black circles) and the Δ*katG* mutant (red triangles). Exogenous H_2_O_2_ (50 μM) was added at 0 s. Catalase-dependent O_2_ evolution in the wild type (blue diamonds) was measured in the dark without adding hydroxylamine. Data are mean ± the standard deviation of three experiments (technical replicates).

**FIGURE 7:**
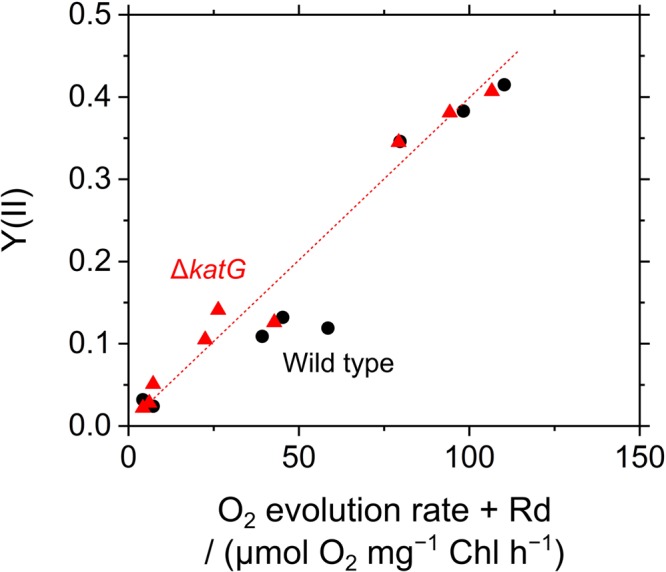
Relationship between the incident quantum yield of PSII, Y(II), and photosynthetic O_2_ evolution rate accelerated by exogenous H_2_O_2_ in *Synechococcus elongatus* PCC 7942. Photosynthetic O_2_ evolution rate is shown as the sum of net O_2_ evolution rate and dark respiration rate (Rd). Data of both Y(II) and O_2_ evolution rate were obtained in (1) phase *α*, (2) phase *β*, and (3) phase *β* with exogenous H_2_O_2_ (50 μM), respectively, in the wild type (black circles) and in the Δ*katG* mutant (red triangles). Experiments were performed three times (technical replicates).

The acceleration of LEF by exogenous H_2_O_2_ was evaluated also by measuring O_2_ evolution rate in *S. elongatus*. Scavenging of H_2_O_2_ by the peroxidase reactions caused O_2_ evolution at PSII, because the regeneration of the reductants is coupled to LEF (v). H_2_O_2_-dependent O_2_ evolution was measured in the presence of hydroxylamine (25 μM), as the activity of catalase in *S. elongatus* is so large that it masks O_2_ evolution derived from the peroxidase reactions (Shimakawa et al., 2017b). Unfortunately, we could not completely inhibit the catalase activity of *S. elongatus* wild type by hydroxylamine at 25 μM. A portion of the catalase-dependent O_2_ evolution rate was detected in the dark even in the presence of hydroxylamine (15 ± 5 μmol O_2_ mg^-1^ Chl h^-1^, mean ± standard deviation, *n* = 3), or approximately 5% of the intact activity (**Figure 6**). To solve this problem, we constructed an *S. elongatus* mutant (Δ*katG*) deficient in the dominant gene encoding catalase (Supplementary Figure S1). In the dark, the O_2_ evolution by the catalase reaction was not observed in Δ*katG* in the presence of hydroxylamine at 25 μM. In the dark, the addition of H_2_O_2_ to the wild type of cyanobacterial cells rapidly induced the evolution of O_2_, indicating instantaneous decomposition of H_2_O_2_ to H_2_O and O_2_ by catalase (**Figure 6**); H_2_O_2_ rapidly entered the cells. However, induced O_2_ evolution proceeded more slowly in the presence of hydroxylamine in both, wild type and Δ*katG*, compared with the catalase reaction (**Figure 6**). The retardation of O_2_ evolution induction reflects the slow relaxation of RISE, which is consistent with the slow decrease in F_s_ and the slow increase in NADPH fluorescence (**Figure 5**), probably due to the low production rate of the oxidized form of electron donors for the peroxidase reaction. We evaluated the relationship of Y(II) to overall O_2_ evolution (the sum of O_2_ evolution rate and dark respiration rate [Rd]) in wild type and Δ*katG* of *S. elongatus*, validating the relationship between scavenging of H_2_O_2_ and LEF (**Figure 7**). Linearity of the relationship was recognized in Δ*katG*, which supported the idea that H_2_O_2_-dependent O_2_ evolution rate reflected peroxidase reaction scavenging H_2_O_2_ coupled to photosynthetic electron transport reactions.

We need to point out that the acceleration of LEF by H_2_O_2_ *via* the peroxidase reaction might not occur as long as the P700 oxidation system and the catalase-dependent H_2_O_2_-scavenging reaction are in operation. Firstly, the production of H_2_O_2_ in PSI would be strictly suppressed where P700 is oxidized. Especially flavodiiron protein dissipates excess photon energy in PSI to prevent the production of superoxide anion radical, which significantly decreases the physiological relevance of H_2_O_2_-dependent LEF in cyanobacteria (Helman et al., 2003; Allahverdiyeva et al., 2013; Weenink et al., 2015; Shimakawa et al., 2016b). Secondly, cyanobacteria show the greater scavenging activity of H_2_O_2_ in the catalase reaction, compared with the peroxidase reaction (**Figure 6**) (Shimakawa et al., 2017b). Therefore, we eliminated the effects of catalase on the cells with hydroxylamine and the mutant Δ*katG* to create the situation where peroxidase-dependent H_2_O_2_-scavenging reactions operate in *S. elongatus*. Overall, in this study, we used the peroxidase reaction in *S. elongatus* as an experimental tool for verification of RISE in phase *β* in *S. elongatus*, because the peroxidase reaction functions with photosynthetic electron transport and the scavenging of H_2_O_2_ does not dissipate ΔpH across the thylakoid membrane for ATP regeneration, but rather, it promotes the formation of ΔpH. That is, RISE can only be relieved by the oxidation of PQH_2_. Conversely, RISE can be induced only by the reduction of PQ to oxidize P700 in PSI.

## Conclusion

In the present research, we showed that RISE functioned on the donor side of PSI to oxidize P700 in wild type cyanobacterium, *S. elongatus*. In phase *β*, P700 in PSI is oxidized in response to suppressed photosynthetic CO_2_ assimilation (**Figure 2C**; Shaku et al., 2016; Shimakawa et al., 2016b). The oxidation of P700 is driven by two mechanisms: (1) acidification of luminal side of the thylakoid membrane (i.e., ΔpH) lowers the oxidation activity of PQH_2_ in Cyt *b*_6_/*f* (Trubitsin et al., 2003; Kramer et al., 2004); and (2) accumulation of PQH_2_ suppresses the Q-cycle in Cyt *b*_6_/*f* to lower the oxidation activity of PQH_2_ (i.e., RISE) (Shaku et al., 2016). Under low CO_2_ in phase *β*, addition of NaHCO_3_ stimulated LEF and caused the reduction of the NADPH pool (**Figure 4**). These results suggest that a donor-side limitation of electron flow in PSI arises, as shown in the oxidation of P700 (Shaku et al., 2016; Shimakawa et al., 2016b). Added NaHCO_3_ relieves the donor-side limitation to enhance electron flux to oxidized P700, leading to NADPH production. Unfortunately, NaHCO_3_-dependent acceleration of LEF cannot be considered conclusive evidence for RISE operating, because stimulated photosynthesis by NaHCO_3_ not only oxidizes PQH_2_ but also dissipates ΔpH. Thus, at this point, we could not exclude the possibility that a ΔpH-dependent control of electron flux from Cyt *b*_6_/*f* to oxidized P700 functions as depicted in **Figure 4**. We therefore continued to determine whether the H_2_O_2_ scavenging reaction stimulated reduction of NADP^+^, in order to elucidate the mechanism of suppressed PQH_2_ oxidation. Some peroxidases, including thioredoxin peroxidase and peroxiredoxin Q, require LEF-supplied NADPH as the electron donor for continuous scavenging of H_2_O_2_ (Yamamoto et al., 1999; Miyake and Asada, 2003; Stork et al., 2009). In other words, the H_2_O_2_ scavenging reaction by peroxidases drives LEF with the formation of ΔpH (Schreiber and Neubauer, 1990; Yamamoto et al., 1999; Miyake and Asada, 2003; Stork et al., 2009). The reduction of NADP^+^ was enhanced by electron flux through LEF upon addition of H_2_O_2_ to *S. elongatus* cells in phase *β* (**Figure 5**); concomitantly, oxidation of PQH_2_ enhanced electron flux to NADP^+^, which strongly supports the idea that RISE is regulated by the redox state of PQ, as reported by Shaku et al. (2016).

## Author Contributions

CM conceived the original screening and research plans and supervised the experiments. GS and KS performed all the experiments. CM, GS, and KS conceived the project, designed the experiments, analyzed the data, and wrote the article.

## Conflict of Interest Statement

The authors declare that the research was conducted in the absence of any commercial or financial relationships that could be construed as a potential conflict of interest.
