# Supplementary material for: Reduction-Induced Suppression of Electron Flow (RISE) Is Relieved by Non-ATP-Consuming Electron Flow in Synechococcus elongatus PCC 7942
Source: Front Microbiol. 2018 May 7;9:886. doi: 10.3389/fmicb.2018.00886 (PMC5949335; doi:10.3389/fmicb.2018.00886)
Supplement: Supplementary file 1 [file Image_1.PDF]

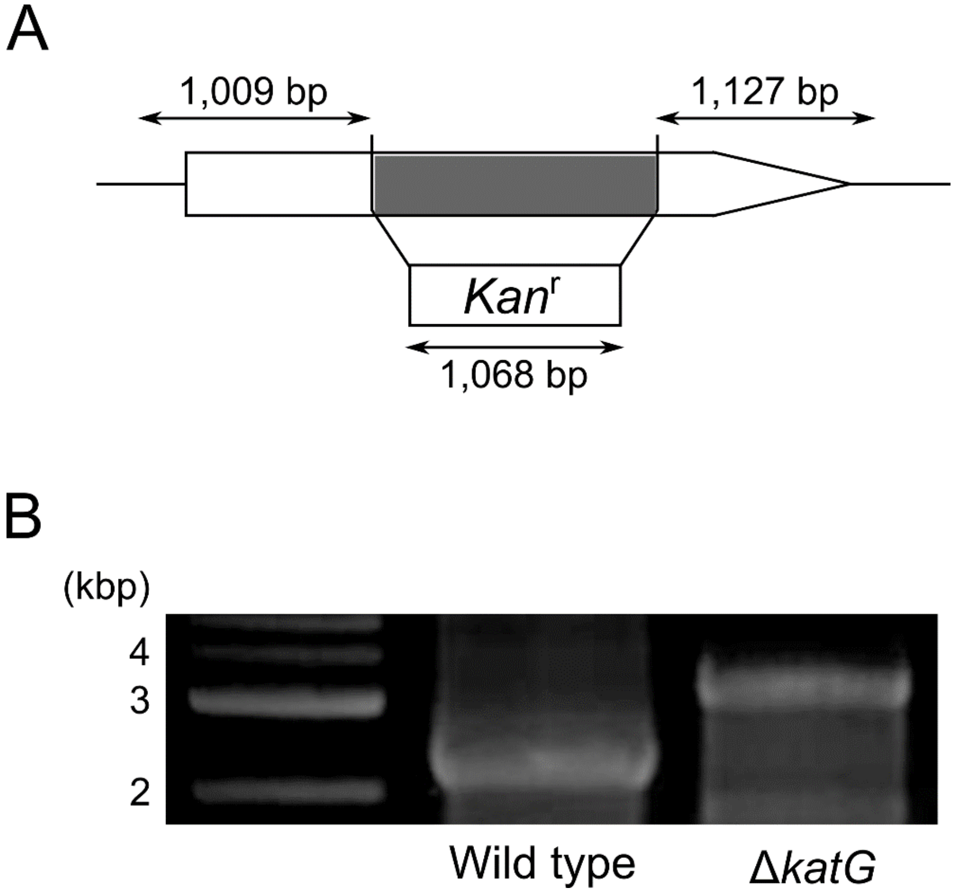

Supplemental Figure S1. Insertional inactivation of *katG* gene in *Synechococcus elongatus* PCC 7942. (A) Physical map of the insertion site of a kanamycin resistance cassette (*Kan*^{r}) in Δ*katG*. The arrowheads indicate the locations of PCR primers. Lengths of the amplified DNA fragments are shown. The full length of the *katG* gene is 2,163 bp. The grey shading indicates the removed genome DNA region (113 bp). (B) DNA fragments amplified by PCR showing complete segregation of the inactivated *katG* gene.

Figure S1. Shimakawa and Shaku et al.
